# Secondary Hematoma Evacuation and Outcome After Initial Conservative Approach for Patients with Cerebellar Hematoma Larger than 3 cm

**DOI:** 10.1007/s12028-021-01203-6

**Published:** 2021-03-02

**Authors:** Sanjula D. Singh, Floris H. B. M. Schreuder, Koen M. van Nieuwenhuizen, Wilmar M. Jolink, Jasper R. Senff, Joshua N. Goldstein, Jeroen Boogaarts, Catharina J. M. Klijn, Gabriel J. E. Rinkel, H. Bart Brouwers

**Affiliations:** 1 Department of Neurology and Neurosurgery, Brain Center Rudolf Magnus, University Medical Center Utrecht, Utrecht, The Netherlands; 2 Division of Neurocritical Care and Emergency Neurology, Department of Neurology, Massachusetts General Hospital, Harvard Medical School, Boston, USA; 3 Department of Neurology, Donders Institute for Brain Cognition and Behavior, Center for Neuroscience, Radboud University Medical Center, Nijmegen, The Netherlands

## Abstract

**Background:**

In patients with spontaneous cerebellar intracerebral hemorrhage (ICH) guidelines advocate evacuation when the hematoma diameter is > 3 cm. We studied outcome in patients with cerebellar ICH > 3 cm who did not undergo immediate hematoma evacuation.

**Methods:**

We included consecutive patients with cerebellar ICH > 3 cm at two academic hospitals between 2008 and 2017. Patients who died < 24 h (h) were excluded because of probable confounding by indication. We determined patient characteristics, hematoma volumes, EVD placement, secondary hematoma evacuation, in-hospital and 3-month case-fatality, and functional outcome.

**Results:**

Of 130 patients with cerebellar ICH, 98 (77%) had a hematoma > 3 cm of whom 22 (23%) died < 24 h and 28 (29%) underwent hematoma evacuation < 24 h. Thus, 48 patients were initially treated conservatively (mean age 70 ± 13, 24 (50%) female). Of these 48 patients, 7 (15%) underwent secondary hematoma evacuation > 24 h, of whom 1 (14%) had received an EVD < 24 h. Five others also received an EVD < 24 h without subsequent hematoma evacuation. Of the 41 patients without secondary hematoma evacuation, 11 (28%) died and 20 (51%) had a favorable outcome (mRS of 0–3) at 3 months. The 7 patients who underwent secondary hematoma evacuation had a decrease in GCS score of at least two points prior to surgery; two (29%) had deceased at 3 months; and 5 (71%) had a good functional outcome (mRS 0–3).

**Conclusions:**

While cerebellar ICH > 3 cm is often considered an indication for immediate hematoma evacuation, there may be a subgroup of patients in whom surgery can be safely deferred. Further data are needed to assess the optimal timing and indications of surgical treatment in these patients.

**Supplementary Information:**

The online version contains supplementary material available at 10.1007/s12028-021-01203-6.

## Introduction

Spontaneous intracerebral hemorrhage (ICH) is the most lethal form of stroke, and its incidence is 10–30 patients per 100,000 persons per year worldwide [1]. Cerebellar ICH accounts for 10% of all ICH with a 30-day mortality varying from 30 to 50% [2–5]. Due to the anatomy of the posterior fossa, life-threatening complications such as hydrocephalus, brainstem compression, and herniation through the foramen magnum can occur [6]. Neurosurgical treatment can be life-saving and is performed more often in cerebellar ICH compared to supratentorial ICH [7].

The European Stroke Organization (ESO) guidelines state that there is inadequate evidence to make a strong recommendation about how, when, and for whom hematoma evacuation of cerebellar ICH should be performed [8]. The American Heart Association (AHA) guidelines advocate for immediate surgery in patients with spontaneous cerebellar hemorrhage with a diameter of > 3 cm or those showing signs of brainstem compression or hydrocephalus [9]. This recommendation is based on small, observational studies [10]. Randomized controlled trials (RCTs) comparing surgical versus non-surgical treatment have not been performed [11, 12].

Therefore, we aimed to investigate clinical variables and outcomes in patients with cerebellar ICH who were initially treated conservatively, to determine in which subgroup of patients and how often, secondary hematoma evacuation was required.

We often refrain from hematoma evacuation in patients who have a cerebellar hematoma of > 3 cm and a Glasgow Coma Scale score (GCS) of 14 or 15, since clinical signs may be more valid predictors of the clinical course and outcome than the diameter of the hematoma. In those with a depressed level of consciousness in whom hydrocephalus (rather than the hemorrhage itself) is thought to be contributing or causing the poor clinical condition, an external ventricular drain (EVD) is often placed to reduce intracranial pressure without a large posterior fossa craniotomy. In case of a low perioperative intracranial pressure as measured via the EVD, the hematoma may be evacuated in the same session (with the patient switched from supine to prone position) if other clinical variables permit.

In this study, we assessed case-fatality and functional outcome in patients with spontaneous cerebellar hemorrhage > 3 cm who survived at least 24 h and received initial conservative treatment or an EVD only rather than immediate hematoma evacuation.

## Methods

### Study Design

We studied a consecutive series of patients with spontaneous cerebellar hemorrhage > 3 cm admitted between December 2008 and July 2017 to the University Medical Center Utrecht (UMCU) or between January 2012 and January 2018 to the Radboud University Medical Center (RUMC), both in the Netherlands. The study was approved by the institutional review boards of both hospitals and was in accordance with institutional guidelines. All analyses were performed retrospectively with prospectively collected de-identified data.

### Study Population

Inclusion criteria were: patients older than 18 years of age with cerebellar hemorrhage diagnosed with either computed tomography (CT) or magnetic resonance imaging (MRI) within 24 h of symptom onset. Exclusion criteria were: (1) patients who died < 24 h of symptom onset; (2) patients in whom a palliative care strategy was implemented < 24 h; (3) patients who underwent hematoma evacuation < 24 h; (4) patients with an underlying vascular lesion, such as arteriovenous malformation, aneurysm, dural fistula, or cavernoma; (5) hemorrhagic transformation of ischemic stroke; (6) ICH caused by neoplasms; (7) traumatic cerebellar ICH.

All patients were treated according to our respective institutional protocols and were admitted on either an intensive or high care unit, according to the clinical condition upon admission. “In the Netherlands, non-intubated patients are observed at a ‘medium or high care’ unit dedicated to neurological and neurosurgical patients where GCS score and vital signs are being monitored as often as at the ICU or even with invasive measures e.g., arterial lines.”

### Clinical Data

For all patients, the following clinical data were collected: age, sex, medication use (including anticoagulation), past medical history, blood pressure on admission, Glasgow Coma Scale score on admission, time between symptom onset to initial CT scan, time between baseline and follow-up CT scan (if performed), presence of hydrocephalus (as reported by neuroradiologists), EVD placement, and (secondary) hematoma evacuation. When follow-up CT scans were available, hematoma expansion (> 33% or > 6 ml), brainstem compression, herniation through the foramen magnum, and tight posterior fossa sign were evaluated. Secondary hematoma evacuation was defined as hematoma evacuation after 24 h. Trained research nurses assessed case-fatality and functional outcome, as measured by the modified Rankin Scale (mRS), at discharge and at 3 months after discharge. The mRS was dichotomized into favorable functional outcome (0–3) versus unfavorable functional outcome (4–6).

### Imaging Analyses

Hematoma volumes were calculated using the ABC/2 methods by two reviewers (S.D.S and H.B.B) independently from each other [13]. Discrepant findings were resolved during consensus measurements, after which consensus was reached.

### Statistical Analysis

Continuous variables are reported as mean and standard deviation (SD) or median and interquartile range (IQR), as appropriate. Discrete variables are expressed as count and proportions. We performed exploratory analyses, limited by the small number of patients who underwent secondary surgery, for baseline characteristics by calculating relative risks of secondary hematoma evacuation with corresponding 95% confidence intervals. We refrained from comparing outcomes between treatment groups, due to confounding by indication and the relatively small number of patients. All statistical analyses were performed using JMP Pro 13 (SAS Institute, Inc). A *p* value of < 0.05 was considered statistically significant, calculated by Fisher’s exact test or Wilcoxon test as appropriate.

## Results

Of 130 patients with spontaneous cerebellar ICH, 98 (77%) had a hematoma > 3 cm. Of these 98 patients, 22 (23%) died or received withdrawal of care < 24 h (h) and were therefore excluded from this study (Table 1). Patients who underwent hematoma evacuation < 24 h were found to have larger hematoma volumes (25 versus (vs.) 14 mL, *p* ≤ 0.001), lower GCS scores upon arrival (12 vs. 14, *p* = 0.008), more likely to have brainstem compression (89% vs. 40%) on admission CT, and were more likely to suffer from hydrocephalus (79% vs. 31%, *p* < 0.001) compared to those patients receiving conservative treatment < 24 h.

**Table 1:** Baseline characteristics and outcome of cohort

Baseline characteristics	Dead < 24 h or withdrawal of care	Hematoma evacuation < 24 h	Conservative treatment
*Total n = 98*	*n (%)*	*n (%)*	*n (%)*
Number of patients (n, %)	22 (23%)	28 (29%)	48 (49%)
Age (mean, SD)	73.1 (13.4)	68.1 (10.9)	69.6 (13.1)
Female sex	15 (68%)	10 (36%)	24 (50%)
Past medical history			
Hypertension	15 (68%)	16 (57%)	23 (48%)
Diabetes mellitus	3 (14%)	5 (18%)	6 (13%)
Hypercholesterolemia	4 (18%)	10 (36%)	10 (21%)
Ischemic stroke	2 (9%)	2 (7%)	4 (8%)
Transient ischemic attack	3 (14%)	3 (11%)	6 (13%)
Previous intracerebral hemorrhage	2 (9%)	1 (4%)	4 (8%)
Myocardial infarction	2 (9%)	0 (0%)	3 (6%)
Atrial fibrillation/flutter	7 (32%)	8 (29%)	7 (15%)
Medication			
Anticoagulation use	9 (41%)	14 (50%)	16 (33%)
Antiplatelet therapy	4 (18%)	6 (22%)	14 (29%)
Antihypertensive therapy	12 (55%)	16 (57%)	28 (58%)
Lipid lowering therapy	3 (14%)	10 (36%)	12 (25%)
Clinical condition on admission [GCS score] (median, IQR)	3 (3–6)	12 (11–13)	14 (12–15)
Intensive care unit (ICU)	7 (32%)	28 (100%)	13 (27%)
CT characteristics			
Baseline ICH volume (median, IQR)	25.0 (16.9–43.5)	25.1 (18.0–33.1)	14.1 (10.0–22.7)
Intraventricular extension	16 (73%)	20 (71%)	20 (42%)
Hydrocephalus	14 (64%)	22 (79%)	15 (31%)
Medical management			
Prothrombin complex concentrate	4 (18%)	11 (39%)	13 (27%)
Vitamin K	2 (9%)	8 (29%)	8 (17%)
Platelet transfusion	1 (5%)	0 (0%)	3 (6%)
Surgical management			
EVD placement < 24 h	3 (14%)	24 (86%)	4 (13%)
EVD placement > 24 h	0 (0%)	0 (0%)	7 (15%)
Length of stay in days (median, IQR)	1 (0–1)	23 (13–32)	15 (7–19)
Outcome at discharge			
Discharge mRS (median, IQR)	6 (6–6)	4 (4–5)	4 (3–4)
mRS < = 3	0 (0%)	4 (14%)	17 (35%)
mRS 4–5	0 (0%)	19 (68%)	28 (58%)
Died during hospitalization	22 (100%)	5 (18%)	3 (6%)
Outcome at 3 months (n = 92: 22, 27 and 46*)			
mRS (median—IQR)	6 (6–6)	4 (3–6)	3 (2–6)
mRS < = 3	0 (0%)	7 (26%)	25 (54%)
mRS 4–5	0 (0%)	12 (44%)	8 (17%)
Death	22 (100%)	8 (30%)	13 (28%)

*CT* computed tomography, *EVD* external ventricular drainage, *GCS* Glasgow Coma Scale score, *ICH* intracerebral hemorrhage, *IQR* interquartile range, *mRS* modified Rankin Scale score, *SD* standard deviation

^*^Two lost to follow-up in the no secondary surgery group

**Fig. 1:**
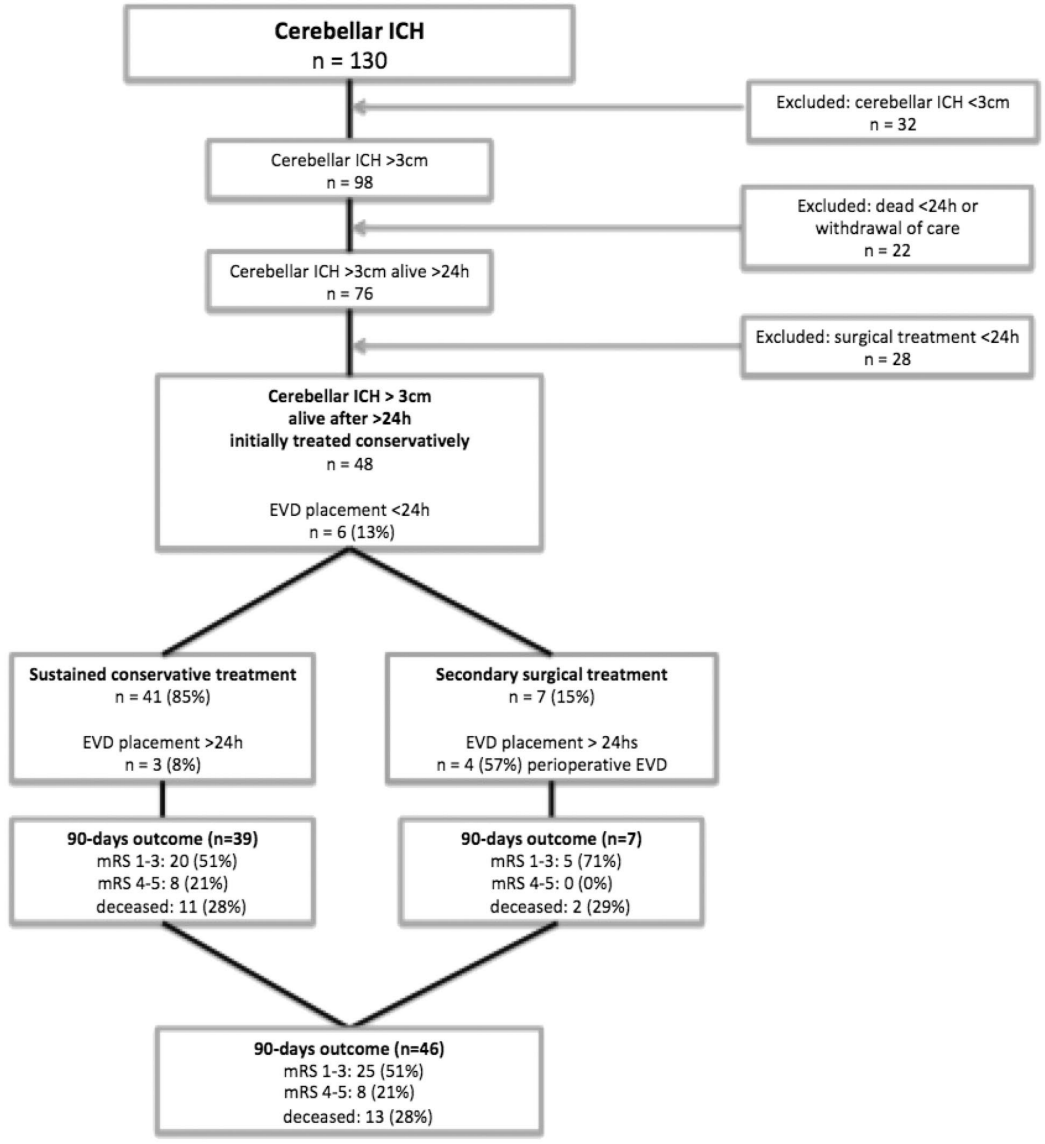
Flowchart. *EVD* external ventricular drainage, *ICH* intracerebral hemorrhage, *mRS* modified Rankin Scale score

Of the remaining 76 patients, 28 (37%) underwent hematoma evacuation within 24 h and thus were also excluded, leaving 48 patients who were included in the study (Fig. 1). Baseline characteristics of these 48 patients are provided in Table 2. Of these 48 patients, 6 patients (13%) received an EVD < 24 h, of which only one (17%) underwent secondary surgery. Of the 41 patients without secondary hematoma evacuation, 3 (7%) underwent EVD placement after 24 h.

**Table 2:** Sustained conservative therapy versus secondary hematoma evacuation

Baseline characteristics	All patients n (%)	No secondary surgery n (%)	Secondary surgery n (%)
Number of patients (n, %)	48	41	7
Age (mean, SD)	69.6 (13.1)	71.2 (11.8)	60.6 (17.6)
Female sex	24 (50%)	21 (51%)	3 (43%)
Past medical history			
Hypertension	23 (48%)	20 (49%)	3 (43%)
Diabetes mellitus	6 (13%)	5 (12%)	1 (14%)
Hypercholesterolemia	10 (21%)	9 (22%)	1 (14%)
Ischemic stroke	4 (8%)	4 (10%)	0 (0%)
Transient ischemic attack (TIA)	6 (13%)	6 (15%)	0 (0%)
Previous intracerebral hemorrhage	4 (8%)	4 (10%)	0 (0%)
Myocardial infarction	3 (6%)	3 (7%)	0 (0%)
Atrial fibrillation/flutter	7 (15%)	7 (17%)	0 (0%)
Medication			
Anticoagulation use	16 (33%)	16 (39%)	0 (0%)
Antiplatelet therapy	14 (29%)	13 (32%)	1 (14%)
Antihypertensive therapy	28 (58%)	25 (61%)	1 (43%)
Lipid lowering therapy	12 (25%)	11 (37%)	1 (14%)
Clinical condition on admission [GCS score] (median, IQR)	14 (12–15)	14 (12–15)	14 (13–15)
Intensive care unit (ICU)	13 (27%)	7 (17%)	6 (86%)
CT characteristics			
Baseline ICH volume (median, IQR)	14.1 (10.0–22.7)	12.7 (9.2–22.0)	
Intraventricular extension	20 (42%)	17 (42%)	3 (43%)
Hydrocephalus	15 (31%)	10 (24%)	5 (71%)
Medical management			
Prothrombin complex concentrate	13 (27%)	13 (32%)	0 (0%)
Vitamin K	8 (17%)	8 (20%)	0 (0%)
Platelet transfusion	3 (6%)	3 (7%)	0 (0%)
EVD placement < 24 h	6 (13%)	5 (12%)	1 (14%)
EVD placement > 24 h	7 (15%)	3 (8%)	4 (57%)
Outcome at discharge			
mRS (median—IQR)	4 (3–4)	4 (3–4)	4 (3–4)
mRS 0–3	17 (35%)	15 (37%)	2 (29%)
mRS 4–5	28 (58%)	23 (56%)	5 (71%)
Died during hospitalization	3 (6%)	3 (7%)	0 (0%)
Outcome at 3 months (n = 46, 39, 7*)			
mRS (median—IQR)	3 (2–6)	3 (2–6)	3 (2–6)
mRS 0–3	25 (54%)	20 (51%)	5 (71%)
mRS 4–5	8 (17%)	8 (21%)	0 (0%)
Death	13 (28%)	11 (28%)	2 (29%)

*CT* computed tomography, *EVD* external ventricular drainage, *GCS* Glasgow Coma Scale score, *ICH* intracerebral hemorrhage, *IQR* interquartile range, *mRS* modified Rankin Scale score, *SD* standard deviation

^*^Two lost to follow-up in the no secondary surgery group

Patients who underwent secondary hematoma evacuation suffered more often from hydrocephalus (71 vs. 24%, *p* = 0.005), were younger (60 vs. 71, *p* = 0.17), and had larger baseline ICH volumes (19 vs. 13 mL, *p* = 0.085) compared to sustained conservatively treated patients. The risk ratios and corresponding 95% CI for secondary hematoma evacuation are shown in Supplementary Table 1.

### Secondary Hematoma Evacuation

Seven patients (15%) underwent secondary hematoma evacuation, who all had a decrease in GCS score of at least two points prior to surgery. Of these seven patients, four (57%) had hematoma expansion on the pre-operative CT scan, six (86%) tight posterior fossa sign, and all brainstem compression and herniation through foramen magnum.

Of the 7 patients with secondary hematoma evaluation none died during hospital admission. At 3 months, 2 had died (case-fatality rate 29%). At discharge 2 patients (29%) and at three months 5 patients (71%) had a good outcome.

### Sustained Conservative Therapy

Of the 41 patients without secondary hematoma evacuation, 30 (73%) underwent a follow-up CT scan of which 7 (23%) presented with hematoma expansion, 14 (47%) with brainstem compression, 9 (30%) with tight posterior fossa sign, and 15 (50%) with herniation through foramen magnum.

Out of these 41 patients, 3 died during hospital admission (case-fatality rate 7%), with follow-up data lacking for 2 patients. Out of the 39 patients with complete follow-up, 11 had died at three months (case-fatality rate 28%). A favorable outcome was reported in 15 (35%) patients at discharge and in 20 patients (51%) at 3 months.

## Discussion

In this cohort, approximately two-third of patients with cerebellar ICH > 3 cm without withdrawal of care < 24 h and with initial conservative treatment is alive at three months, and half of them have a good functional outcome. The proportion of patients undergoing secondary hematoma evacuation is small (7 out of 48, 15%), and this proportion is similar for patients with or without EVD insertion within the first 24 h.

Although confounding by indication inevitably plays a role in our study, an initial conservative approach was opted for in most patients with a cerebellar ICH > 3 cm. Thus, it is a substantial subgroup of all patients with cerebellar ICH that may benefit from an initial conservative approach. This even holds in the subgroup of patients (with a hematoma > 3 cm) for which some of the guidelines currently advice differently. Similar findings were reported in previous literature, which emphasized that clinical condition, rather than hematoma size solely, predicts outcome in patients with cerebellar ICH who were treated with hematoma evacuation. Therefore, hematoma evacuation was not recommended in patients with cerebellar ICH with favorable initial neurological condition [14].

In this study, we demonstrated that patients who underwent hematoma evacuation < 24 h at our two institutions, were found to have larger hematoma volumes, lower GCS scores upon arrival, more often suffered from hydrocephalus and were more often admitted to the ICU (Table 1). No differences were found between institutions, and analyses between physicians were not available. In patients who were initially treated conservatively but received hematoma evacuation > 24 h, neurological deteriorating was an important factor in determining whether to perform secondary hematoma evacuation (Table 2).

Several limitations of this study should be taken into consideration. First and foremost, we cannot compare results from the initial conservative approach with those with initial hematoma evacuation, due to confounding by indication. It is unknown whether the outcome of patients would have been better if all patients with cerebellar ICH > 3 cm had undergone initial surgical treatment. We did not systematically collect extensive clinical data during hospitalization, and therefore, we were unable to use these data as potential determinants of secondary hematoma evacuation. Moreover, we did not systematically perform follow-up CT scans after 24 or 48 h. In addition to this, the relative risks with corresponding 95% confidence intervals, as displayed in Supplementary Table 1, were determined in exploratory analyses and should be interpreted with caution, as six risk factors were taken into consideration, but only seven patients underwent secondary surgery. Lastly, we were unable to compare our data with previous literature, as we did not find any previous studies specifically focusing on the initial conservative treatment and secondary hematoma evacuation of patients with spontaneous cerebellar hematomas larger than 3 cm. However, a review on associations between hematoma evacuation and outcome presented data on limited improvement in functional outcome after surgery, subsequently stressing the need of subgroup analyses to determine evidence-based treatment criteria and guidelines [15].

The strengths of this study are a relatively large cohort of patients from two academic hospitals. Moreover, we had a very low rate of missing data (all variables had a missing percentage of < 10%, and > 90% had a missing percentage of < 5%). As the available literature is insufficient to guide treatment decisions in cerebellar ICH, this study adds compelling new data to this debate.

In conclusion, the present study demonstrates that an initial active conservative approach often leads to good outcome in patients with a cerebellar hematoma > 3 cm, thus making this a reasonable option. Whether this approach is superior or inferior to immediate surgical evacuation of a cerebellar hematoma > 3 cm in patients in a good clinical condition should be evaluated in large, prospective registries and eventually in a randomized controlled trial.

## Supplementary Information

Supplementary file 1 (DOCX 16 KB)
