# Supplementary material for: Secondary Hematoma Evacuation and Outcome After Initial Conservative Approach for Patients with Cerebellar Hematoma Larger than 3 cm
Source: Neurocrit Care. 2021 Mar 2;35(3):680–6. doi: 10.1007/s12028-021-01203-6 (PMC8692294; doi:10.1007/s12028-021-01203-6)
Supplement: Supplementary file 1 — (DOCX 16 KB) [file 12028_2021_1203_MOESM1_ESM.docx]

**Supplementary Table 1. Risk Ratios of secondary hematoma evacuation**

| Risk factor | Risk Ratio (95% CI) |
| --- | --- |
| Age (>71, median) | 0.8 (0.3-2.0) |
| Antiplatelets | 0.5 (0.1-2.9) |
| Baseline volume (>14, median) | 1.5 (1.0-2.3) |
| Intraventricular extension | 1.0 (0.5-2.6) |
| Hydrocephalus | 2.9 (1.4-6.0) |
| EVD placement <24 hours | 1.2 (0.2-8.6) |

EVD = External Ventricular Drainage
